# Supplementary material for: Patterns of Intron Gain and Loss in Fungi
Source: PLoS Biol. 2004 Nov 30;2(12):e422. doi: 10.1371/journal.pbio.0020422 (PMC532390; doi:10.1371/journal.pbio.0020422)
Supplement: Table S1 — Also available at http://genes.mit.edu/NielsenEtAl/. (4.3 MB ZIP). [file pbio.0020422.st001.zip › NielsenEtAl/html/929.html]

AN0560.1.NCU06631.1.MG06098.1.FG09816.1


```
 CLUSTAL W (1.82) Multiple Sequence Alignments - Introns Inserted


Sequence 1: NCU06631.1	621 aa
Sequence 2: MG06098.1	680 aa
Sequence 3: FG09816.1	679 aa
Sequence 4: AN0560.1	666 aa
Alignment Length: 721 aa
Number Identitical Residues: 219 aa
Alignment Score (without introns) 12208


MG06098.1 	MSDERGLKSLRAGAKRKTVRPTISNPRPVNEPN---KNVDIPRGRAGPPDGPLVVRQRPP
NCU06631.1	MSEERSKISLRSGGKRK-NRPTISAPRQISAPIPQDNGMPQPPPEATMADEPRMTRPRPP
FG09816.1 	-MEDRNKISLRSGRRKK--RPTISAPRQISGPIAQDDSNRPPLPGADPSQASAQPRPRPP
AN0560.1  	-MDGRG-LTLRSKNRRP--RPQISAPKPISGPLP--QNHQPAASGSGTASSGSGSRDYAS
          	  : *.  :**:  ::   ** ** *: :. * .  ..   .   :  ..     *  ..

MG06098.1 	-LASGK0TSDLVKRRFSTRFNDLPTDGFAP---PMPALPSFDSYEAASSRDREMRGAPPS
NCU06631.1	-PPGGK0TSDLVKKRYSTRFGNMPTD-FDPSSNPMPALPDLEKYMQAQVGSPPSRGGDAG
FG09816.1 	PMAGGK0TSDLVKRRYSTRFN-QPSSISNGGAPPMPQMPSLANYEPRET-VAAARKPPSR
AN0560.1  	--SNHA~TSDLVKRRYSTRFNQVPDFDGAP---PVPSVPQVPSAYAGLGPPQPSRKQSAE
          	  ..   ******:*:****.. *         *:* :*.. .           *   . 

MG06098.1 	RGAG----APLSVDRRVLGDPNLSAEQY~VAKILSDATEDEIREFEESLRGLKDGTSQRL
NCU06631.1	-GLGPMGREPPRVDIRTLRDPNFAPEQY1VAEVLGEATEDEIRDYEDALKQLKARAAADL
FG09816.1 	SGPG----VAPVIDIKGLRDPKLKPDRY~VQAALSDATEDQIREFEESLRQVKTRVGTDL
AN0560.1  	-SSG-----PPEVDLTALRDPSLPVDRY1VTNLLANASEDDIREYQQALRKVKNRTSTDL
          	 . *     .  :*   * **.:  ::* *   *.:*:**:**:::::*: :*  ..  *

MG06098.1 	QANVLQNRTQFIKISKEAEKLKGEMRALKNLMSELKANTTALRAASSGNGPNGDSSDPSG
NCU06631.1	QQNVYQNRTQFIKISKEAEKLKGEMRTLRNLMLELKTNTTALRASSNSADSSG-SLGP--
FG09816.1 	QQSVMQNRTQFIKISKEAEKLKSEMRNLKNFMSELKVNTTAMRAAAAKSDEPMPSDLG--
AN0560.1  	QQNVYQNRTQFIRISQEADKLKGEMKTLRSLMAEL---TTALGQTAIGDSPNPMSPTL--
          	* .* *******:**:**:***.**: *:.:* **   ***:  ::   .    *     

MG06098.1 	GFSTGLS-KRDKRSSVADRTALWNSQMQ~ALYKNVEGSQKFLPHSPTRHVLQNAGPWIEL
NCU06631.1	EFSTGLS-KRDRRSSITDRTALWSAQMQ0ALYKSVEGSQKFLPNSQGRHVVQNAGPWIEL
FG09816.1 	-VAPGISSRRDRRTSIADRSAMWNSQMQ~ALYKGVEGSQKFLPNAVGRHVVQDAGPWIEL
AN0560.1  	--DERAS-KRSNRSSVANLESMWNVQLQ~TLWKTVEGSQKFLPMVPGRHIVLETGNWAEL
          	      * :*..*:*:::  ::*. *:* :*:* *********    **:: ::* * **

MG06098.1 	DNATYKSRRSIQIFLLNDHLLIASRKKRKADVSG-DFRGPE-MKLVADRCWPLLDIEVVD
NCU06631.1	DNATYKSRRSMQIFLLNDHLLIASRKKRKIDGPGADARGPM-TKLVADRCWHLLDVEVVD
FG09816.1 	DNATYKSRRAMQIFLLNDHLLIASRKKRKADAPGADARGPM-MKLVADRCWPLLDVEVVD
AN0560.1  	DSATWKPRRPVHLVLLNDHLLVAAKKRKRVDQSNPNHRGPVPTKLIAEECWPLQDIDMID
          	*.**:*.**.:::.*******:*::*::: * ...: *** . **:*:.** * *::::*

MG06098.1 	MSG--SGETSS-GRNK-LAEAIMVRGVGQ-ETLIYRTEKPDGPEKASLLMNIRKAVEELR
NCU06631.1	MAG--TGDSSN-GRNK-LADAIMVRGGGQNESFIYRTEKPEDPEKTTLILNIRKTVEELR
FG09816.1 	MSS--TGESSNSGRNK-LADAIMVRGVGQ-ESFIYRTEKPQDPEKKQLLLNVRKAIEQLR
AN0560.1  	LGANLTGSAREEAEDRGITNAVCVR-VGS-KPFTYRHDKRNSTAKSELLATFRKTVEDLR
          	:... :*.: ....::.:::*: **  *. :.: ** :* :.. *  *: ..**::*:**

MG06098.1 	RGLQSEMDANNKAKDTINYFASRDPG-------LLQKTALLETLSDIKDMLIEVDGKQQN
NCU06631.1	RNLQSERDATNKAKETINYFASRDPG-------LLQKTELLETLSDIKDMLIEVDGKQQN
FG09816.1 	KGLRSEMEANNKARETINYFASRDPG-------LLQKTELLATLSDIKDMLIEVDGKQQN
AN0560.1  	RTLRSETEAAGKNGESLGFMSAINSRNSLLCSPKLDLSENTDNPRDRPEVRIDVDGKQQN
          	: *:** :* .*  :::.:::: :. .:  .:. *: :    .  *  :: *:*******

MG06098.1 	LRWVESQMDELDIDVALQRFDPAVQRVEKLKGLARGLKNNAIAQDFIEFKVEERTAKLAA
NCU06631.1	LRWVESQMDELDINIALQQIEPAVARIEMLKNLASGLKNNMIAQDFISFKVDERCARLAT
FG09816.1 	LRWVESEMDDLDIDVAMQRFEDAVVRVEKLKAIARGLKNHAIAQDFINFKVNERCVRLAN
AN0560.1  	LRWVDSQVDELDIDIALQRFEEAVSNIDRLRKLARGLKGNAVAQDVINTKVDERAAKLAG
          	****:*::*:***::*:*::: ** .:: *: :* ***.: :***.*. **:** .:** 

MG06098.1 	IISRELVDSHDSPRKTKRNVAWLTKLGFEDRAREAYLEARSNIIQKRSR2QCIFQGDLWV
NCU06631.1	LVVRELVNSHNDQKKTKRNVTWLVRLGFEDRAREAYLEARSEVIQKRS-~----------
FG09816.1 	MIGRELEMTHDNNTKTRRNVSWLTRLGFEDSARESYLAARSGTIHKRTR2QCIFQGDLHL
AN0560.1  	ILSRSLVDTHGFPVATKTNVVWLTRLGFEDQARESYLKARSDVISKRIR2----------
          	:: *.*  :*.    *: ** **.:***** ***:** ***  * **             

MG06098.1 	YIWEISFVYFTVIKNTVACFQSCFPPPMMS~VCVKWAKEEVDAFNAILARQLSSTERDGE
NCU06631.1	-----------------------------S2ACVKWAKEEVDAFNVILARQLSSAEEGGE
FG09816.1 	YIWELSFVYFMVIHNTVQCFQSCFPPPMMS~VCVKWAKEEVDAFNIILARQLSSTELRGQ
AN0560.1  	------------------TYQQCFPSVMTS~ACIKWAKHHLDAFNALLTRQLSSVQRGAT
          	                    ... ..   * .*:****..:**** :*:*****.:  . 

MG06098.1 	~IWRKCVDQAKTHADMLSEVGLDFRNLVGRVPEP----GAAKKAASQAPALGLGLS----
NCU06631.1	0AS------MGIGTDSGPEAERAFR---ARYSDR----GD--------------------
FG09816.1 	~VWTQCMERAKEHSKLLSEVGLDFENLVGKNLMI----YEP---IDQGPSVGLGLS----
AN0560.1  	~VWQNCINIVHEQAGILAEVGVDFTDLVAKELELTEEEKAARPEMTRSESLIMGLADAAS
          	    .. .     :   .*.   * .  .:    :...  .      . :   . :.:::

MG06098.1 	~-------
NCU06631.1	~-------
FG09816.1 	~-------
AN0560.1  	1FFNMYGH
          	   .  .
```
